# Artificial Intelligence–Augmented Clinical Decision Support Systems for Pregnancy Care: Systematic Review

**DOI:** 10.2196/54737

**Published:** 2024-09-16

**Authors:** Xinnian Lin, Chen Liang, Jihong Liu, Tianchu Lyu, Nadia Ghumman, Berry Campbell

**Affiliations:** 1 School of Education Fuzhou University of International Studies and Trade Fuzhou China; 2 Department of Biomedical Informatics and Medical Education School of Medicine University of Washington Seattle, WA United States; 3 Department of Health Services Policy and Management Arnold School of Public Health University of South Carolina Columbia, SC United States; 4 Department of Epidemiology and Biostatistics Arnold School of Public Health University of South Carolina Columbia, SC United States; 5 Department of Obstetrics and Gynecology School of Medicine University of South Carolina Columbia, SC United States

**Keywords:** artificial intelligence, biomedical ontologies, clinical decision support systems, implementation science, obstetrics, pregnancy, AI, systematic review, CDSS, functionality, methodology, implementation, database query, database queries, bibliography, record, records, eligibility, literature review, prenatal, early pregnancy, obstetric care, postpartum care, pregnancy care, diagnostic support, clinical prediction, knowledge base, therapeutic, therapeutics, recommendation, recommendations, diagnosis, abnormality, abnormalities, cost-effective, surveillance, ultrasound, ontology

## Abstract

**Background:**

Despite the emerging application of clinical decision support systems (CDSS) in pregnancy care and the proliferation of artificial intelligence (AI) over the last decade, it remains understudied regarding the role of AI in CDSS specialized for pregnancy care.

**Objective:**

To identify and synthesize AI-augmented CDSS in pregnancy care, CDSS functionality, AI methodologies, and clinical implementation, we reported a systematic review based on empirical studies that examined AI-augmented CDSS in pregnancy care.

**Methods:**

We retrieved studies that examined AI-augmented CDSS in pregnancy care using database queries involved with titles, abstracts, keywords, and MeSH (Medical Subject Headings) terms. Bibliographic records from their inception to 2022 were retrieved from PubMed/MEDLINE (n=206), Embase (n=101), and ACM Digital Library (n=377), followed by eligibility screening and literature review. The eligibility criteria include empirical studies that (1) developed or tested AI methods, (2) developed or tested CDSS or CDSS components, and (3) focused on pregnancy care. Data of studies used for review and appraisal include title, abstract, keywords, MeSH terms, full text, and supplements. Publications with ancillary information or overlapping outcomes were synthesized as one single study. Reviewers independently reviewed and assessed the quality of selected studies.

**Results:**

We identified 30 distinct studies of 684 studies from their inception to 2022. Topics of clinical applications covered AI-augmented CDSS from prenatal, early pregnancy, obstetric care, and postpartum care. Topics of CDSS functions include diagnostic support, clinical prediction, therapeutics recommendation, and knowledge base.

**Conclusions:**

Our review acknowledged recent advances in CDSS studies including early diagnosis of prenatal abnormalities, cost-effective surveillance, prenatal ultrasound support, and ontology development. To recommend future directions, we also noted key gaps from existing studies, including (1) decision support in current childbirth deliveries without using observational data from consequential fetal or maternal outcomes in future pregnancies; (2) scarcity of studies in identifying several high-profile biases from CDSS, including social determinants of health highlighted by the American College of Obstetricians and Gynecologists; and (3) chasm between internally validated CDSS models, external validity, and clinical implementation.

## Introduction

In the United States, maternal and newborn outcomes (eg, maternal and newborn mortality, preterm birth, low birth weight, congenital abnormalities, and maternal pregnancy complications) are worse than in any other resource-rich countries, where most pregnancy-related mortalities were preventable [1]. Severe maternal mortality and morbidity has led to significant short- or long-term consequences impacting not only pregnant individuals but also their families [2]. Across the health care system in the nation, there remains a limited number of pregnant individuals who have access to evidence-based, comprehensive, and continuous maternity care [1]. Maternity providers often experience insufficient information and limited guidelines to inform clinical decisions, in part because studies conducted in small sample sizes and pregnant and lactating individuals are generally excluded from clinical trials [3].

To address the chasm, the clinical decision support system (CDSS) has broad application in pregnancy care. In the clinical practice guideline provided by the American College of Obstetricians and Gynecologists, the role of CDSS was described along the pathway of patient management and was increasingly exposed within emerging clinical information systems such as electronic health records (EHRs) [4]. Because pregnancy care often is characterized by multi-modal (eg, clinical findings, medical imaging, noninvasive prenatal testing, and genomics), multi-specialty data (eg, obstetrics, maternal-fetal medicine, gynecology, reproductive endocrinology and infertility, and neonatology), and complex episodes (ie, from preconception, conception, prenatal, intrapartum, to post partum), precise and timely clinical decision support requires a very high level of EHR interoperability and clinical validity. Within the context of this study, pregnancy care is defined as the health care for mothers and fetus (or newborn) before, during, and after the pregnancy. In addition to improving patient care management, CDSS also plays a critical role in supporting evidence-based medicine and the pathway toward a learning health system, in which CDS bridges the gap between the increasingly available digital data and much-demanded actionable knowledge for therapeutics and patient care [5,6].

The recent evolution of artificial intelligence (AI) and biomedical informatics has led to new frontiers in clinical and translational medicine. Within the scope of this study, we refer to the definition of AI in health care broadly as the methods and applications that create computer systems capable of activities normally associated with cognitive effort during health care [7]. In the field of CDSS, the application of AI has become increasingly prominent in augmenting knowledge discovery, diagnostics support, risk prediction and alarming, chronic disease management, and patient monitoring, to name a few [8]. In pregnancy care, emerging research has been exploring how AI-augmented CDSS would help improve clinical workflow and patient management. However, with the vast number of clinical guidelines, diverse AI techniques, and different EHR systems and functional modules, the spectrum of capacities and characteristics that such AI-augmented CDSS would further improve pregnancy care remains unclear. Increasing numbers of AI studies in obstetrics and gynecology have been documented [9-11]. However, reviews on how AI-augmented CDSS were specialized in pregnancy care have been missing.

A systematic review is desperately needed to fill several gaps in the literature. First, maternal health decisions are preference-sensitive and have been based on limited evidence-based guidelines. Such characteristics require the CDSS to be designed taking into account both the existing clinical guidelines, carefully selected clinical data, and shared patient consent or preference data. The dynamic of these CDSS designing features appears differently in specific episodes and subspecialty of pregnancy care, which needs to be reviewed systematically yet no existing reviews have achieved this goal. Second, recent AI applications on CDSS for pregnancy care appear to be different from those from one decade ago. Although the concept of AI remains loosely defined, a systematic review is desired to update the state-of-the-art AI methodologies applied to pregnancy-related CDSS, as well as to provide a timely comparative evaluation against those historical AI technologies. Third, existing literature reviews of either AI applications or CDSS in the field of pregnancy care typically focus on the evaluation of various AI methods and the performance of a model (eg, prediction, classification, and information retrieval). No studies have evaluated model performance together with implementation assessment outcomes of the CDSS in real-world settings, which has been a missing perspective of the external validity of CDSS studies.

The objective of this study is to provide a systematic review of empirical studies that examined AI-augmented CDSS in pregnancy care and inform challenges and opportunities with respect to how findings from these emerging studies would improve pregnancy care using the framework of participants, interventions, comparisons, outcomes, and study design as reference. Specifically, we sought to (1) identify specific maternal care domains where AI-augmented CDSS plays a role, (2) characterize the current state of CDSS functions, and (3) identify limitations, challenges, and future opportunities.

## Methods

The literature review follows the PRISMA (Preferred Reporting Items for Systematic Reviews and Meta-Analyses) [12]. The PRISMA checklist can be found in Multimedia Appendix 1.

### Bibliographic Database

We searched 3 electronic bibliographic databases: PubMed/MEDLINE (including MEDLINE and PubMed Central [PMC]), Embase, and ACM Digital Library. PubMed/MEDLINE is a bibliographic database of life sciences and biomedical topics established by the US National Library of Medicine at the National Institutes of Health in 1996. PMC was launched in 2000 as a digital counterpart to the National Library of Medicine’s extensive print full-text journal collection of biomedical and life sciences. Some PMC journals are cross-indexed as MEDLINE journals. Embase is a bibliographic database focused on pharmacovigilance. The ACM Digital Library is a comprehensive database of full-text studies and bibliographic literature covering computing and information technology including biomedical informatics and digital health. Licenses for accessing Embase and ACM Digital Library are obtained by the University of South Carolina.

### Search Strategy

In the PubMed/MEDLINE database, we searched strings in the field of “text word,” which includes “title, abstract, other abstract, Medical Subject Headings (MeSH) terms, MeSH subheadings, publication types, substance names, personal name as subject, corporate author, secondary source, comment or correction notes, and other terms.” To supply topics mis-captured or were not precisely captured by “text word,” we also searched string in [MeSH Major Topic]. We adjusted the search strategy used for PubMed/MEDLINE in Embase and ACM Digital Library, respectively. We modified the search fields for Embase and ACM Digital Library because MeSH is only used in PubMed/MEDLINE and other fields vary in the three bibliographic databases. For all databases, the time of publications was constrained to be including and before 2022. No language restrictions were applied. The search strings mainly incorporated pregnancy procedures, pregnancy outcomes, CDSS models, CDSS methods, and AI methodologies (see Multimedia Appendix 2 for search strings and criteria). All electronic reference database searches were completed in January 2023. The search strategy was developed by two authors (CL and TL) with consolidated suggestions received from other authors. The search was performed by NG.

### Assessment of Eligibility and Biases

**Figure 1:**
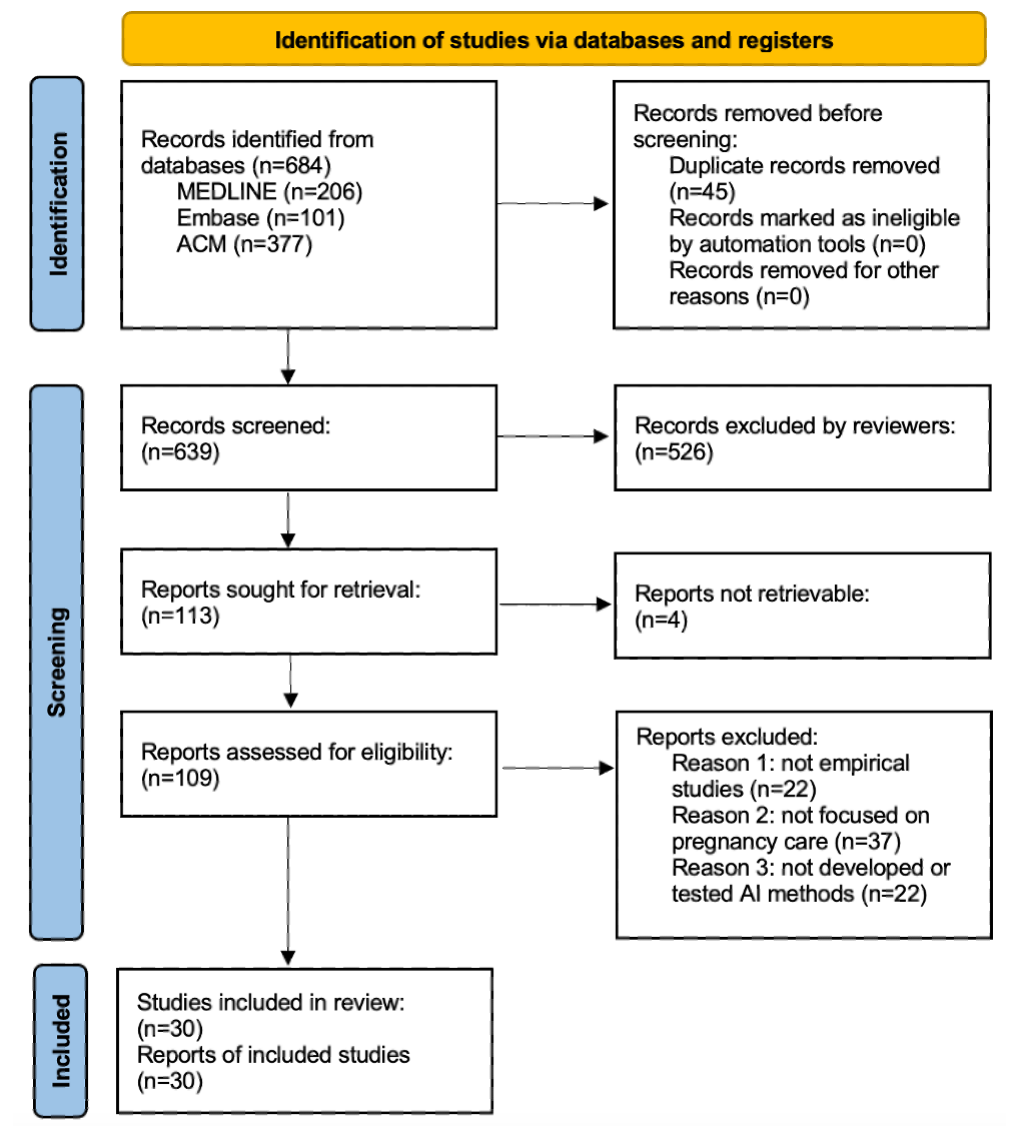
PRISMA flowchart. AI: artificial intelligence; PRISMA: Preferred Reporting Items for Systematic reviews and Meta-Analyses. Note: Under “Reports excluded,” exclusion reasons #1, #2, and #3 are not mutually exclusive.

One author (NG) removed duplicates when comparing results from each database. In this process, PubMed Identifier, titles, publications, and authors are used to identify unique publications. To perform the eligibility assessment, we used the following inclusion criteria: empirical studies that (1) developed or tested AI methods, (2) developed or tested CDSS or CDSS components, and (3) focused on pregnancy care. Quality and biases of studies were assessed based on several criteria adopted from the Risk of Bias 2 tool [13], which include (1) whether an empirical study, (2) concentration of “pregnancy care,” “CDSS,” and “AI” in the study, and (3) completeness, clarify, and validity of methods, results, and conclusion as reported in the publications. Full-text manuscripts of potentially relevant studies were reviewed for final inclusion. Following these criteria, two reviewers (TL and NG) independently inspected the candidate publications for inclusion and quality. Discrepancies between the two reviewers were resolved through discussion with a senior reviewer (CL) and then, corrected and finalized. Finally, there were 30 studies selected for review (see Figure 1 for the study selection process).

### Data Synthesis

When authors reported ancillary information (eg, pilot study), or overlapping outcomes of a study reported from different publications, we grouped such publications as one single unit of publications. Two independent coders (CL and TL) extracted the following study information: authors and year, study objectives, pregnancy care applications, CDSS functionality, data source, study population, AI methods, CDSS performance, validation, and implementation. Pregnancy care applications included three categories: prenatal and early pregnancy care, obstetric care, and postpartum care. In the context of this review, obstetric complications include maternal (eg, perinatal hemorrhage, ectopic pregnancy, eclampsia, and gestational diabetes), fetal (eg, miscarriage, stillbirth, and preterm birth), and neonatal (eg, bradycardia and tachyarrhythmia) adverse events. In the context of this review, obstetric complications include maternal (eg, perinatal hemorrhage, ectopic pregnancy, eclampsia, and gestational diabetes), fetal (eg, miscarriage, stillbirth, and preterm birth), and neonatal (eg, bradycardia and tachyarrhythmia) adverse events. Of note, these three categories are not mutually exclusive. With respect to CDSS functionality, the definition of clinical prediction refers to the prediction of adverse clinical events, outcomes, prognosis, and identification of at-risk individuals with adverse events. With respect to types of validation, internal validation refers to the process of validating the performance of CDSS models inside the context of the study, by which the context of the study means the model training set and testing set are partitioned from the same data set that has a high degree of homogeneity (eg, from the same clinical site and same patient cohort). Internal validation emphasizes on validity of model accuracy, as well as the sample size. External validation refers to the process of validating the performance of CDSS outside the context of the study, which empathizes with the generalizability of the internally validated CDSS to and across other contexts (eg, clinical sites, patient cohorts, times, and data quality).

## Results

### Study Selection and Synthesis of Results

**Figure 2:**
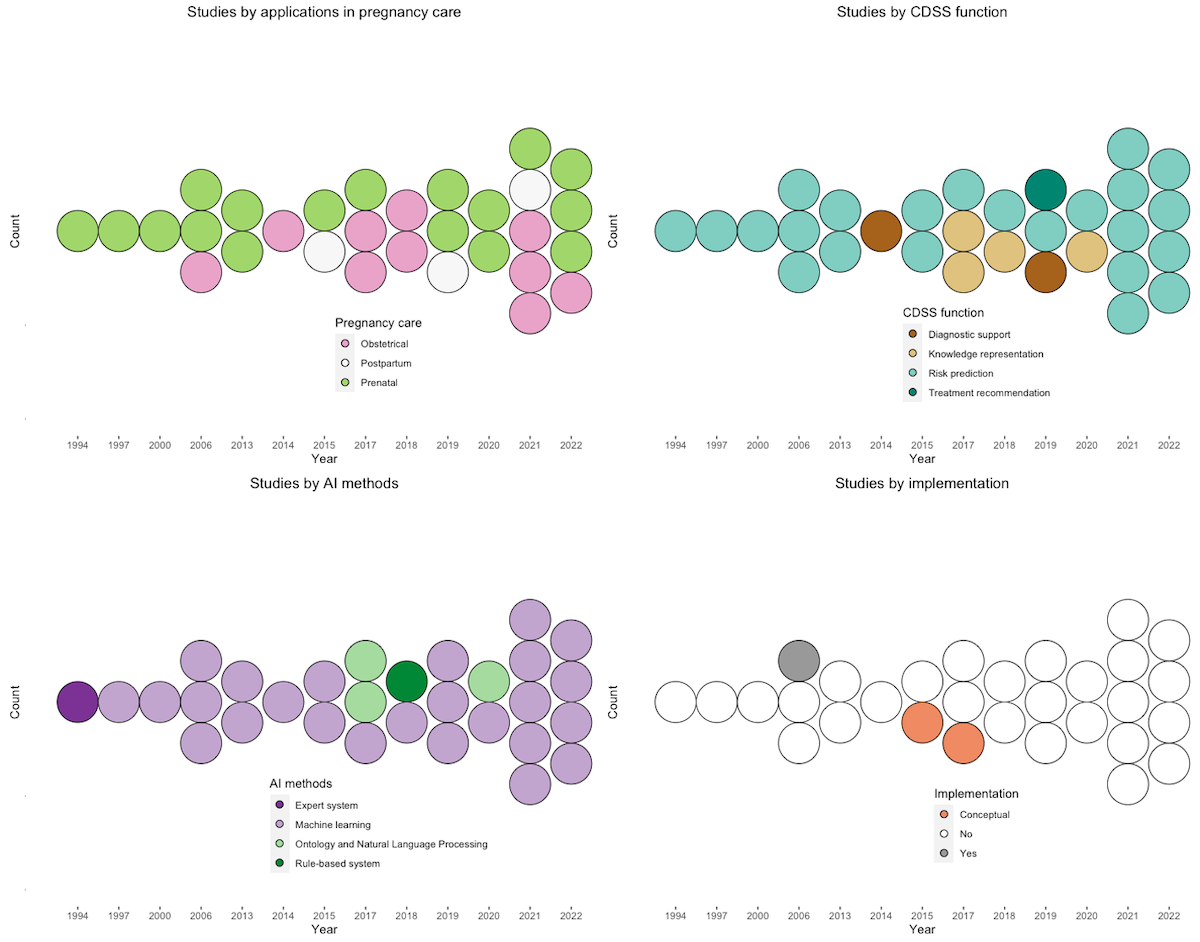
Trends in reviewed studies. Top-left: trends in studies by applications in pregnancy care. Top-right: trends in studies by CDSS function. Bottom-left: trends in studies by AI methods. Bottom-right: trends in studies by implementation. AI: artificial intelligence; CDSS: clinical decision support system.

We included 206 studies from PubMed/MEDLINE, 101 studies from Embase, and 377 studies from ACM Digital Library. Removal of studies hit by exclusion criteria and duplicates resulted in 30 distinct studies that met the eligibility criteria (Figure 1). We analyzed the 30 studies and characterized findings into structured themes, summarized in Table 1. Over time, the number of relevant studies has increased except for a dip in 2013-2014 (Figure 2).

**Table 1:** Summary of reviewed studies.

Study	Study objectives^a^	CDSS^b^ functions	Data source	Sample	AI^c^ methods	Performance	Validation	Implementation
Woolery and Grzymala-Busse (1994) [14]	Expert system for *preterm birth risk assessment*.	Risk prediction	Registry (multiple sites, United States)	18,890 cases	Expert system, machine learning	ACC^d^ 53%-88%	External	No
Mongelli et al (1997) [15]	Develop an expert system for the interpretation of *fetal scalp acid-base status*.	Risk prediction	Scalp blood samples (single, England)	2174 samples	Logistic transformations, back-propagation networks, decision tree	N/A^e^	Internal	No
Goodwin et al (2000) [16]	Predict *preterm birth*.	Risk prediction	EHR^f^ (single, United States)	19,970 patients	Rule induction, logistic regression, neural network	Customized (AUC^g^ 0.75)	Internal	No
Catley et al (2006) [17]	*Obstetrical outcome estimations* in low-risk maternal populations.	Risk prediction	Registry (37 sites, Canada)	48,000 cases	ANN^h^	ROC^i^ 0.73	Internal	No
Mueller et al (2006) [18]	Identify *predictors to optimize* *extubation decisions for premature infants*.	Risk prediction	EHR (single, United States)	183 infants	ANN, multiple layer regression	AUC >0.9	Internal	Yes
Gorthi et al (2009) [19]	Predict *pregnancy risk* based on patterns from clinical parameters.	Risk prediction	Synthetic cases	200 cases	Decision tree	ACC 82.5	Internal	No
Ocak (2013) [20]	Assess *fetal well-being*.	Risk prediction	Cardiotocogram (single, United States)	1831 samples	SVM^j^	ACC 99.3%	Internal	No
Yılmaz and Kılıkçıer (2013) [21]	Determine the *fetal state* using cardiotocogram data.	Risk prediction	Cardiotocogram (single, United States)	2126 samples	LS-SVM^k^	ACC 91.62%	Internal	No
Spilka et al (2014) [22]	Examine cardiotocogram and support decision-making (*outcomes: diagnostics and risk*).	Diagnostic support	Cardiotocogram (single, United States)	634 samples	Latent class analysis	N/A	Internal	No
Jiménez-Serrano et al (2015) [23]	Detect the *postpartum depression* during 1st week after childbirth. Toward a mobile health app.	Risk prediction	Registry (7 sites, Spain)	1880 women	Logistic regression, naïve bayes, SVM, ANN	ANN (ACC 0.79)	Internal	Conceptual
Ravindran et al (2015) [24]	Assess *fetal well-being*.	Risk prediction	Cardiotocogram (single, United States)	2126 samples	Ensemble: k-NN^l^, SVM, Bayesian network, and ELM^m^	ACC 93.61%	External	No
Paydar et al (2017) [25]	Predict *pregnancy outcomes* among systemic lupus erythematosus-affected pregnant women.	Risk prediction	EHR (single, Iran)	149 pregnant women	MLP^n^, RBF^o^	MLP (ACC 0.91)	Internal	Conceptual
Dhombres et al (2017) [26]	Develop a knowledge base for ectopic pregnancy.	Knowledge representation	Ultrasound (single, England)	4260 records	Ontology, NLP	Precision 0.83	Internal	No
Maurice et al (2017) [27]	Develop a new knowledge base intelligent system for ultrasound imaging.	Knowledge representation	PubMed (single, United Kingdom)	N/A	Ontology, NLP	F 0.71	Internal	No
Fergus et al (2018) [28]	Classify *cesarean section and vaginal delivery*.	Risk prediction	Registry (single, Czechia)	552 pregnancies	Ensemble: RF, SVM, decision tree, ANN, deferred acceptance	Ensemble (AUC 0.96)	Internal	No
Seitinger et al (2018) [29]	Arden Syntax as medical knowledge representation and processing language in obstetrics.	Knowledge representation	N/A	N/A	Arden syntax	N/A	N/A	No
De Ramón Fernández et al (2019) [30]	Develop a decision support system to make *suggestions for early treatment for ectopic pregnancy*.	Treatment recommendation	EHR (single, Spain)	406 tubal ectopic pregnancies	Multilayer perception, decision rule, SVM, Naïve Bayes	SVM (ACC 0.96)	Internal	No
Wang et al (2019) [31]	Develop a *postpartum depression* prediction model using EHR.	Risk prediction	EHR (single, United States)	179,980 pregnancies	Logistic regression, SVM, decision tree, Naïve Bayes, XGB^p^, RF^q^	SVM (AUC 0.79)	Internal	No
Liu et al (2019) [32]	Predict *pregnancies*.	Diagnostic support	Mobile app	65,276 women	Logistic regression, LSTM^r^	AUC 0.67	External	No
Ye et al (2020) [33]	Predict *GDM*^s^ and compare their performance with that of logistic regressions.	Risk prediction	EHR (single, China)	22,242 singlet pregnancies	Gradient Boosting Decision Tree, AdaBoost, LightGBM, logistic regression, voting, XGB, decision tree, RF, logistic regression^t^	GBDT^u^ (AUC 0.74, 95% CI 0.71-0.76)	Internal	No
Silva et al (2020) [34]	Develop readable and minimal syntax for a web CDSS for antenatal care guidelines.	Knowledge representation	N/A	N/A	Ontology	N/A	No	No
Venkatesh et al (2021) [35]	Predict *the risk of postpartum hemorrhage at labor admission*.	Risk prediction	EHR (Consortium on Safe Labor, United States)	228,438 deliveries	RF, XGB, logistic regression^t^, lasso regression^t^	XGB (C statistic0.93; 95% CI 0.92-0.93)	External (multi-site, multi-time)	No
Tissot and Pedebos (2021) [36]	Test embedding strategies in performing risk assessment of miscarriage before or during pregnancy.	Risk prediction	EHR (InfoSaude, Brazil)	4676 pregnancies	Machine learning, ontology embedding	KRAL^v^ (F 0.76)	Internal	No
Escobar et al (2021) [37]	Predict *risk of maternal, fetal, and neonatal events*.	Risk prediction	EHR (15 sites, United States)	303,678 deliveries	Gradient boosted, logistic regression^t^	Gradient boosted (AUC 0.786)	External	No
Tao et al (2021) [38]	Construct a hybrid *birth weight* predicting classifier.	Risk prediction	EHR (single, China)	5759 pregnant women	LSTM, CNN^w^, RF, SVM, BPNN^x^, logistic regression	Hybrid LSTM (MRE^y^ 5.65 ± 0.4)	Internal	No
Mooney et al (2021) [39]	Examine RF to predict the *occurrence of hypoxic-ischemic encephalopathy*	Risk prediction	Registry (2 sites, Sweden)	53,000 deliveries	RF	RF (MCC^z^ 0.63)	Internal	No
Du et al (2022) [40]	Predict *gestational diabetes mellitus*.	Risk prediction	Registry (single, Ireland)	565 women	XBG, AdaBoost, SVM, RF, logistic regression	SVM (AUC 0.79)	Internal	No
Schmidt et al (2022) [41]	Predict *adverse outcomes in patients with suspected preeclampsia*	Risk prediction	Ultrasound (single, Germany)	1647 patients	Gradient Boosting Decision Tree, RF	GBTree (AUC 0.81)	Internal	No
De Ramón Fernández et al (2022) [42]	Predict *mode of delivery*: cesarean section, eutocia vaginal delivery, instrumental vaginal delivery.	Risk prediction	Registry (single, Spain)	10,565 records	MLP, RF, SVM	ACC >90	Internal	No
Hershey et al (2022) [43]	Predict *spontaneous preterm birth*.	Risk prediction	Surveys, biospecimen (10 centers)	2390 women	SVM	AUC 0.75	Internal	No

^a^The outcomes of a CDSS model are given in italics.

^b^CDSS: clinical decision support system.

^c^AI: artificial intelligence.

^d^ACC: accuracy.

^e^Not applicable.

^f^EHR: electronic health record.

^g^AUC: area under the receiver operating characteristic curve.

^h^ANN: artificial neural network.

^i^ROC: receiver operating characteristic curve.

^j^SVM: support vector machine.

^k^LS-SVM: least-squares support vector machine.

^l^k-NN: k-nearest neighbors.

^m^ELM: extreme learning machine.

^n^MLP: multilayer perceptron neural network.

^o^RBF: radial basis functions neural network.

^p^XGB: XGBoost.

^q^RF: random forest.

^r^LSTM: long-short term memory.

^s^GDM: gestational diabetes.

^t^Benchmark algorithm

^u^GBDT: gradient-boosted decision tree.

^v^KRAL: knowledge representation and artificial learning.

^w^CNN: convolutional neural network.

^x^BPNN: back propagation neural network.

^y^MRE: mean relative error.

^z^MCC: Matthew’s correlation coefficient.

### Risk of Bias of Included Studies

**Figure 3:**
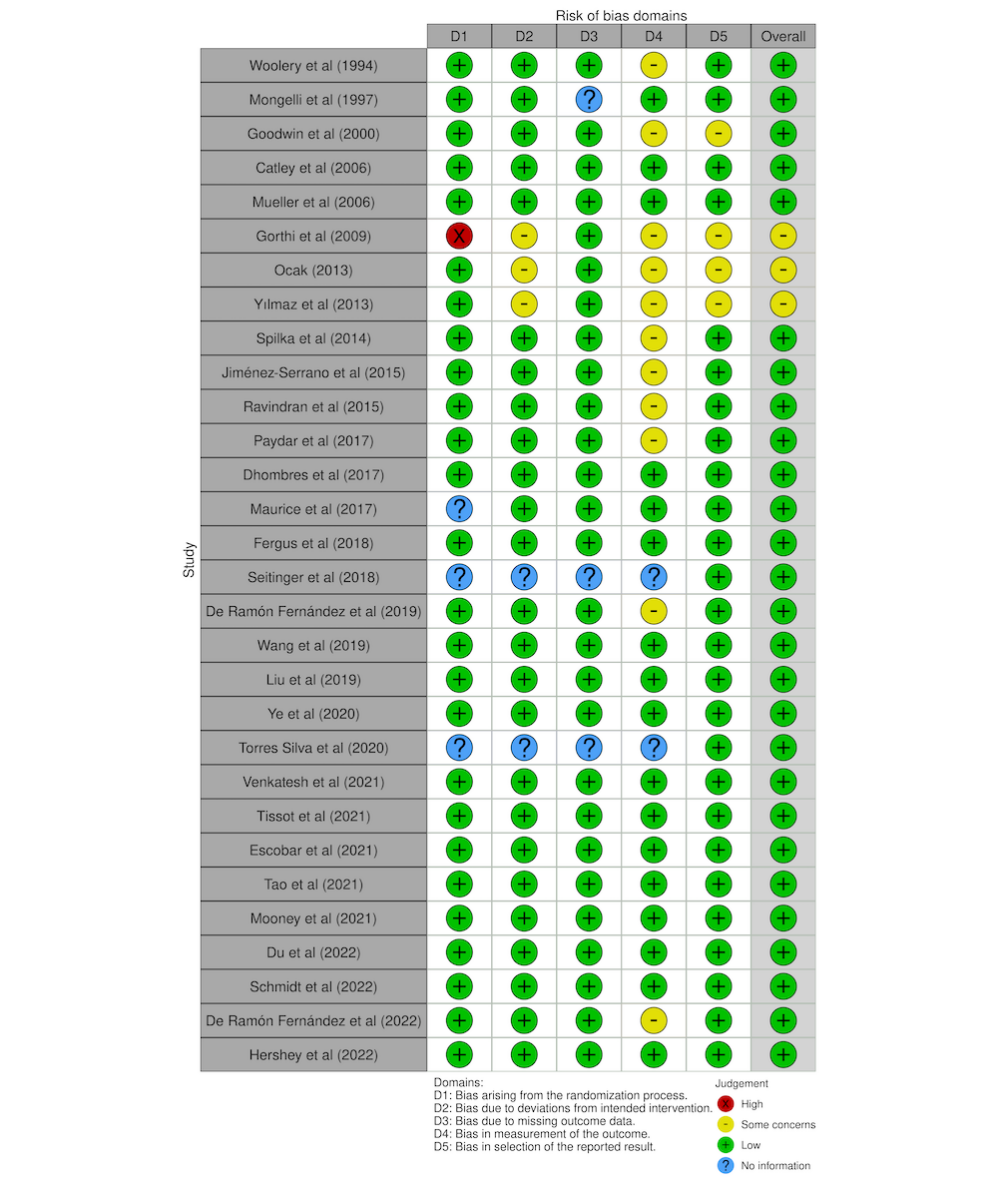
Traffic light plot for risk-of-bias assessment of included studies.

Among the 109 studies screened and retrieved, we excluded 22 studies that are not empirically based, 37 studies that pregnancy care is not the primary focus, and 22 studies that do not develop or apply AI methods even though AI-related terms are widely used in the publications. This appraisal process resulted in 30 included studies that have reached a full agreement of quality between the two reviewers (TL and NG) after discussing with the third reviewer (CL). See Figure 1 for the numbers of included and excluded studies from every step. Following the PRISMA guidelines for assessing the risk of bias of included studies, we summarized the assessment outcome in Figure 3.

### Study Characteristics: Applications in Pregnancy Care

#### Prenatal and Early Pregnancy Care

Detection of maternal and fetal risk factors and abnormalities during prenatal care is most imperative for timely prevention and intervention (n=17, 57%). Studies using CDSS include the prediction of gestational diabetes mellitus [33,40], miscarriage [25,36], and adverse outcomes resulting from preeclampsia [41] using data from medical history and prenatal care visits. Another use case of CDSS has applied to ectopic pregnancy which is a highly risky condition that often leads to maternal morbidity and mortality [30]. Upon diagnosis, choosing adequate treatment is an important clinical decision process to avoid further complications. Machine learning–based CDSS has been tested to aid providers and patients in better-informed clinical decision-making following ectopic pregnancy [30].

#### Obstetrical Care

As the number of delivering individuals who experience morbidity and mortality remains high and growing in the United States and many countries [44], an increasing volume of CDSS studies have been focusing on developing predictive models for early detection of adverse events and among at-risk individuals to be used for timely prevention and intervention (n=10, 33%). For example, the identification of individuals with risks of preterm birth can inform advanced medical care planning at the prenatal and perinatal stages [14,17,43]. These studies generally included analyses of risk factors contributing to adverse events, in which machine learning–based studies rely on feature ranking methods for the identification of data highly suggestive of adverse outcomes. Another example is computer-assisted cardiotocography (CTG) trace interpretation to be used before or at labor and delivery to assist decision-making [21,22,28].

#### Postpartum Care

CDSS has been used for estimating the risk of postpartum hemorrhage at labor and delivery admission (n=3, 30%). Postpartum hemorrhage has been a major source of maternal morbidity and mortality, accounting for nearly one-third of deaths of birthing individuals [45]. Risk estimation used in clinical practice has been based on stratification of risk factors documented in individuals’ medical records using parametric statistic models. In recent CDSS studies, researchers have attempted to incorporate nuances beyond known risk factors [35], which may better interpret individual variance, and reduce possible biases from traditional guidelines and theoretical frameworks. Another example is risk assessment and screening of postpartum depression, which is a prevalent postpartum disorder but is often underdiagnosed [23,31].

### Study Characteristics: Functionality of CDSSs

#### Diagnostic Support

Diagnostic support is a classic function known since early stage CDSS (n=2, 7%) [46]. In pregnancy care, this function has been used to assist the interpretation of CTG [21,22,28] because CTG interpretation is known to be challenging due to a great interreviewer variability and accurate interpretation of CTG is important for making proper clinical decisions during prenatal care and labor and delivery (eg, cesarean vs vaginal delivery). Diagnostic support has also been applied for the identification of pregnancy using data collected from mobile devices [32], which may have value for family planning and preventive care.

#### Clinical Risk Prediction

Clinical prediction as a CDSS function was not prevalent at the inception of CDSS and has started to emerge in pregnancy care recently (n=22, 73%). In pregnancy care, risk prediction tools were broadly used for early detection of adverse maternal and fetal events. Such CDSS applications have been centered around making the prediction of adverse events that may benefit from early detection of abnormalities for timely prevention and intervention, such as eclampsia or preeclampsia [37,41], gestational diabetes [33,40], preterm birth [14,17], miscarriage [25,36], perinatal hemorrhage [35,37,41], hypoxic-ischemic encephalopathy [39], low birth weight [38], and postpartum depression [23,31]. EHR and medical images were often used for training predictive models. A few studies used data from mobile apps [23,32].

#### Therapeutics Recommendation

Mode of delivery is one of the critical clinical decisions to make during obstetric care (n=2, 7%). Over the last decades, cesarean delivery has been increasing and was found to be associated with increased adverse fetal outcomes, as well as adverse maternal outcomes in subsequential childbirth deliveries [47]. CDSS studies have tested the feasibility of using machine learning to make suggestions out of three delivery modes: cesarean section, eutocic vaginal delivery, and instrumental vaginal delivery [42].

#### Knowledge Base

CDSS can be categorized as those built on top of a knowledge base and those independent of a knowledge base (n=4, 13%). Several studies reported the design and construction of a knowledge base that can underpin CDSS specialized for pregnancy care. Among these studies, forms of knowledge base include Arden syntax and ontology that have been widely used for formal representation of clinical guidelines and graph-based medical knowledge, as well as XML as a markdown language for web and mobile-based CDSS applications. Specifically, in support of diagnostics and therapeutics recommendations for ectopic pregnancy, ontology has been used for supporting the annotation of medical images (eg, ultrasound images for obstetrics) [26,27]. Arden syntax was used to formalize obstetric clinical guidelines into a knowledge base that supports CDSS functions for obstetrics [29]. XML was used for encoding a knowledge base that underpins mobile app–based CDSS for prenatal care [34].

### Study Characteristics: AI Methodologies and Applications

#### Algorithms

Knowledge-base–independent CDSS typically rely on computational algorithms for learning about decision boundaries, whereas supervised algorithms (eg, classification, prediction, and association rules learning) require human-annotated data as a gold-standard sample whereas unsupervised algorithms (eg, clustering) find decision boundaries without a gold standard. In this review, regression-based algorithms were widely used as benchmark algorithms for clinical prediction tools, diagnostic support, and therapeutants recommendation. Some of the studies used parametric linear statistical models as benchmarks [35]. Among supervised machine learning algorithms, support vector machine, random forest, and gradient boosting algorithms (eg, XGBoost) have been increasingly adopted and have revealed outstanding performance. Simple neural networks (eg, multilayer perceptron and artificial neural networks) have been tested as well especially when the feature space of the model is not overly large in dimensionality and complex [17,18,30]. To incorporate domain-specific medical knowledge and human-curated clinical guidelines into machine learning models, the embedding of ontology was also used in the field of pregnancy care [36]. Additionally, there was the application of deep learning algorithms (eg, convolutional neural network and recurrent neural network) in the field which has resulted in trained models outperforming other algorithms in comparison [38].

Knowledge-base–dependent CDSS typically rely on rules (eg, if-then and fuzzy logic) or semantic relations (eg, semantic properties defined by ontology). For example, natural language processing (NLP) tasks (eg, named entity recognition and semantic reasoning) were applied in conjunction with the ontology-based knowledge base for the annotation of medical images [26,27]. Studies of rule-based algorithm applications also demonstrated feasibility and robust clinical interpretability [16]. With respect to knowledge base design in these CDSS studies, ontology was commonly used to construct a knowledge base (n=2, 7%) [26,27].

#### Performance Evaluation

The majority of the studies (n=28, 93%) have tested internal validation to some degree. Validation frameworks used in reviewed studies included hold-out, n-fold cross-validation, and bootstrap or cross-validation. Evaluation metrics used include metrics derived from the theory of information retrieval such as precision, recall, *F* measure, area under the receiver operating characteristics curve [48]; metrics based on probabilistic statistics such as mean squared prediction error, Matthew’s correlation coefficient, chi-square, and c-index; metrics based on descriptive statistics such as accuracy; and customized accuracy measures. A few studies used a validation design that allowed for the computing of confidence intervals [33,35], which enhanced the interpretative capability of validation. There were 5 (16%) studies that included external validation [14,24,32,35,37]. These studies generally tested CDSS on separate data sets including those from different clinical sites.

#### Treatment With Possible Bias

Biases in data sampling, data processing (eg, tackling missing data and data normalization), machine learning model training, validation, and algorithm design could lead to deviated performance and actionable clinical decisions resulting from CDSS. Arguably, because clinical decisions are driven by untrained data, ill-sampled training and validation data are seen to bias the AI-augmented systems, with which CDSS could be one example yet such an issue has not been addressed in the existing studies. Without recognizing or understanding biases in samples, appropriate imputation methods could also omit or amplify biases. Upon review, we did not find comprehensive treatment and discussion for remediating possible biases during the design and development of CDSS.

### Study Characteristics: CDSS Implementation

Implementation of CDSS is the final step to incorporate research findings into routine practice. Only a few studies have discussed the conceptual ideas or pilot study design of clinical implementation of reported CDSS (n=3, 10%) [18,23,25]. For implementation studies in a clinical setting, web-based data entry and graphical result presentation were developed for CDSS implementation [18,25]. One study demonstrated the interface of CDSS based on an Android system [23]. We did not see a comprehensive CDSS implementation study design (eg, usability testing) among the reviewed studies.

## Discussion

### Principal Findings

Over the last decades, we have seen the proliferation of AI applications in clinical and translational medicine. However, AI-augmented CDSS has not been systematically reviewed in the field of obstetrics and gynecology. In this study, we assessed related studies by their health care applications, CDSS functionality, AI methodology, and clinical implementation with the goal of providing the state of the art of studies, as well as summarizing advantages, limitations, and possible future directions. Overall, we have identified 30 related studies published between 1994 and 2022 with an upward trend. There was a notable increase starting in 2021. All studies used data from EHR, registry, and mobile devices, except for those focusing on developing knowledge bases for CDSS. In the field of pregnancy care, functions of existing CDSS include diagnostic support (ie, imaging support), clinical prediction, therapeutics recommendation, and knowledge base. A list of traditional CDSS functions was not seen in the field of pregnancy care, including patient safety (eg, alarms for drug-drug reactions and allergies and computerized provider order entry support), clinical management (eg, point-of-care alters, info button, prompts for vaccination, outreach, and referral), and administrative management (eg, assisted medical coding and documentation) [49-51]. Architectures of CDSS include both knowledge-based dependent and independent, in which ontology remains a primary form for constructing a computerizable knowledge base and was often used jointly with NLP methods (eg, named-entity recognition and semantic reasoning). For CDSS that do not rely on a knowledge base, machine learning algorithms (primarily supervised algorithms including simple neural networks and deep learning) were widely used for learning from empirical data and producing or replicating actionable clinical knowledge. Existing studies confirmed that machine learning, ontology, and NLP have been increasingly applied for modern CDSS in the field of pregnancy care.

### Clinical Implication

In the review of the potentials and challenges for adopting existing AI-augmented CDSS for pregnancy care, there are a few aspects. First, well-performed model in individual pregnancy episodes. Existing CDSS have been designed to assist prenatal care, obstetrics, and postpartum care. The majority of the CDSS studies in prenatal care are focused on assisting in the prediction of risks such as miscarriage [36], ectopic pregnancy [26,30], gestational diabetes [33,40], preterm birth [14,16,17], and severe maternal mortality and morbidity events during the prenatal episode [41]. Among predictive models of these CDSS studies, reported predictive performance is generally well. However, we noticed it is often misinterpreted in these studies with respect to how early before an adverse event a CDSS can reliably detect the risk and offer clinical decisions, which is an obvious obstacle before these CDSS models can be used for real-world practice. For obstetrics, CDSS studies have explored ways to assist in making choices of delivery mode [28,42], extubating decisions for preterm infants [18], and diagnostics during birth and delivery [22]. These applications appear to have the potential to be tested, improved, and adopted in clinical settings. In postpartum care, CDSS applications have performed well in assisting postpartum hemorrhage [52] and depression risk detection [23,31]. When tested and adopted in the real-world scenario, effective data collection could be challenging because of limited patient encounters and access to mobile apps post partum.

Second, model interoperability. Aside from the model performance, interoperability is critically important as it determines the degree clinicians can interpret the model output and make sense of contributing factors and nuances a clinical decision is driven. The knowledge base of CDSS is the most interpretable because any knowledge piece is traceable and can be reasoned in different semantic logics in the knowledge base. Our review has found four studies that used biomedical ontology and semantic web techniques to develop the knowledge base for pregnancy risk, antenatal guidelines, ultrasound imaging, and ectopic pregnancy [26,27,34,36]. With respect to CDSS that function as predictive models, two categories were found among the reviewed studies. Parametric models, including regression models [16,18,23,31-33,37,38,40,52], decision trees [15,19,28,31,33,41], shallow neural networks [17,18,23-25,28,30,42], and expert systems [14,15] can easily reveal decision logic, determinants of a decision, as well as their odds to clinicians. Nonparametric models and deep neural networks [20,21,28,30,38,42,43] have the advantage of taking large and high-dimensional data but have limited capability of making the decision mechanism explicit for clinicians.

In addition to the performance and use-case scenario and clinical interpretability of the CDSS, we noticed that data availability and quality have been a pertinent hurdle for developing CDSS for pregnancy care. The reasons are in part unique for pregnancy care in that during prenatal care data are often generated from either a hospital, outpatient obstetrics group, or outpatient laboratory, where different laboratory technologies and protocols of data entry are the norm. Different initiation time and frequency of prenatal care is another cause of unevenly collected patient data for CDSS training, in which individuals with late initiation and low frequency are generally associated with worse outcomes [53], yet this inequality is underestimated in the experimental phase of CDSS design and testing leading to possible bias when adopting CDSS for real-world practice.

### Strengths, Limitations, and Future Directions of Reviewed Studies

#### Overview

Our review has revealed several strengths of CDSS design in pregnancy care. However, because the application of AI-augmented CDSS in the field remains in its infancy, we also identified a handful of limitations followed by suggestions and future directions.

#### Toward Robustness in Internal Validation Design

The review of existing studies exhibited several strengths in study design. (1) Existing CDSS experiments were generally based on real-world EHR, registry, and mobile device data, in which several studies have used data from multiple clinical centers [14,17,23,37,39,43]. While the majority of studies have sufficient sample sizes, a few studies used relatively small samples (n=100~300, see Table 1). (2) The majority of the studies (n=19, 73%), excluding knowledge base studies, have tested multiple AI algorithms for comparison with a selected benchmark. (3) Cross-validation or hold-out methods were generally used for internal validation. (4) Majority of studies measured *F* score (including precision and recall), area under the receiver operating characteristics curve, and metrics derived from probabilistic statistics for model performance. A few studies used accuracy alone, which may not be sufficient for a fair performance validation. Overall, reported model performance is acceptable (see Table 1).

#### Clinical Plausibility

(1) The majority of studies explicitly stated the clinical use scenarios for the reported CDSS, where the capability of early detection of abnormalities, at-risk pregnancies, and risk factors was generally recognized as the core clinical significance of CDSS. Yet, due to the scarcity of prenatal data and difficulties in integrating longitudinal and cross-specialty medical records, early diagnosis and prediction have been limited by data. (2) Existing studies also reflected the challenges of diagnostics and therapeutics recommendations unique to pregnancy care. Such examples include the interpretation of CTG and choosing a delivery mode. However, medical interpretation, such as CTG, historically has shown great variance in interrater reliability, which warrants repeated evaluations for CDSS design. Additionally, there have been controversial discussions pertaining to how the choice of cesarean delivery would adversely affect the fetus and maternal outcomes for subsequential pregnancies [47]. No CDSS studies have considered fetal outcomes or maternal outcomes of subsequential pregnancies for choosing delivery mode in the present pregnancy, limiting the clinical value of this line of applications. With respect to clinical scenarios where AI-augmented CDSS has been applied, we noticed a dearth of research and testing sites in emergency care, which could be interesting to explore in the future.

#### Possible Biases

The use of CDSS could introduce biases on several occasions. (1) Similar to AI algorithms applied in general clinical practice, bias could be from sampling the data to be used for training and testing CDSS. (2) Racial and ethnic disparities have been well documented in a wide spectrum of maternal mortality and morbidity [54]. For example, using patients sampled from wealthy (or poor) neighborhoods to train a CDSS would introduce bias in their behavior or clinical decision prediction. To mediate, design and clinical implementation of CDSS used for pregnancy care should consider targeted populations and their social determinants of health (SDOH). American College of Obstetricians and Gynecologists has made recommendations for patient screenings to enhance the inclusion of SDOH, avoid stereotyping, acknowledge various forms of racial or ethnic discrimination, and improve clinical decision-making that addresses SDOH [55]. However, existing studies have not included the aforementioned considerations and strategies to reduce biases, which are warranted to be set as future directions.

#### External Validation and Implementation

Despite brief discussions around conceptual ideas, external validation and implementation were rare in the reviewed CDSS studies. Without external validation, the clinical usability of these CDSS when generalized to different patient cohorts, health care systems, and times remains undertested. Challenges for external validation and implementation include substandard interoperability of CDSS models and clinical information systems where a CDSS could be implemented. Additionally, the implementation of CDSS would require organizational commitment, localized workflow, usability testing, and staff training to be successful [56]. Because of the proliferation of machine learning for CDSS, the generalizability of machine learning models has become a new challenge. Future directions are suggested to address these identified knowledge gaps and challenges.

### Limitations of This Study

This review has limitations. Our search strategy is mainly based on keywords and MeSH terms, which may not be comprehensive for capturing CDSS studies that did not explicitly use CDSS-related terminologies. Because the notion of CDSS is often loosely defined, we recognize it as a limitation to eligibility criteria in this review. Despite limitations, our results are timely and pertinent which could be used to guide evidence-based clinical practice and future directions for CDSS studies in the field of pregnancy care. This review study also adheres to the PRISMA guidelines and employed two independent reviewers for study selection and evaluation.

### Conclusions

This review summarized state-of-the-art AI-augmented CDSS methods and applications in the field of pregnancy care. This review highlights the proliferation of machine learning-based clinical predictive models and computer-aided diagnostics and therapeutics with acceptable internal validity tested. Recent advances in this line of research include (1) CDSS design targeted for early diagnosis of prenatal abnormalities and early detection of at-risk pregnancies for timely prevention and intervention; (2) challenging medical image interpretation and decision-making that could use the assistance of CDSS; and (3) several knowledge bases needed for specific domains of pregnancy care including, but not limited to, image annotation, adverse events, and clinical guidelines. Future directions are suggested to address possible biases introduced by using AI and CDSS, comprehensive study on external validity and clinical implementation, and continued improvement of clinical plausibility of CDSS.

## Appendix

Multimedia Appendix 1 PRISMA (Preferred Reporting Items for Systematic Reviews and Meta-Analyses) checklist.

Multimedia Appendix 2 Search strings.

## Abbreviations

AI: artificial intelligence
CDSS: clinical decision support systems
CTG: cardiotocography
EHR: electronic health record
MeSH: Medical Subject Headings
NLP: natural language processing
PMC: PubMed Central
PRISMA: Preferred Reporting Items for Systematic Reviews and Meta-Analyses
SDOH: social determinants of health
