# Supplementary material for: Artificial Intelligence–Augmented Clinical Decision Support Systems for Pregnancy Care: Systematic Review
Source: J Med Internet Res. 2024 Sep 16;26:e54737. doi: 10.2196/54737 (PMC11443205; doi:10.2196/54737)
Supplement: Multimedia Appendix 2 [file jmir_v26i1e54737_app2.docx]

**1. PubMed/MEDLINE**

Search string:

*(pregnancy[Text Word] OR Fetus[Text Word] OR Infant[Text Word] OR Newborn[Text Word] OR baby[Text Word] OR pregnant [Text Word] OR maternal[Text Word] OR preterm[Text Word] OR stillbirth[Text Word] OR miscarriage [Text Word] OR abortion[Text Word] OR fetal[Text Word] OR birth[Text Word] OR labor[Text Word] OR perinatal[Text Word] OR prenatal[Text Word] OR antenatal[Text Word] OR gestational[Text Word] OR gestation[Text Word] OR postpartum[Text Word] OR Obstetrics[Text Word] OR Gynecology[Text Word] OR genital[Text Word] OR reproductive[Text Word] OR Pregnancy[MeSH Major Topic] OR Pregnancy Outcome[MeSH Major Topic] OR Fetus[MeSH Major Topic] OR Infant[MeSH Major Topic] OR Infant, Newborn[MeSH Major Topic] OR Neonatal Screening[MeSH Major Topic] OR Infant Mortality [MeSH Major Topic] OR Fetal Death[MeSH Major Topic] OR Infant Death[MeSH Major Topic] OR Perinatal Death[MeSH Major Topic] OR Perinatal Mortality[MeSH Major Topic] OR Abortion, Habitual[MeSH Major Topic] OR Embryo Loss[MeSH Major Topic] OR Fetal Viability[MeSH Major Topic] OR Prenatal Diagnosis[MeSH Major Topic] OR Postpartum Period[MeSH Major Topic] OR Depression, Postpartum[MeSH Major Topic] OR Prenatal Care[MeSH Major Topic] OR Premature Birth[MeSH Major Topic] OR Infant, Premature[MeSH Major Topic] OR Obstetric Labor, Premature[MeSH Major Topic] OR Infant, Extremely Premature[MeSH Major Topic] OR Infant, Extremely Low Birth Weight[MeSH Major Topic] OR Gynecology[MeSH Major Topic] OR Obstetrics[MeSH Major Topic] OR Fetal Monitoring[MeSH Major Topic] OR Pregnancy Complications[MeSH Major Topic] OR Maternal Mortality[MeSH Major Topic]) AND (clinical decision support[Text Word] OR decision aid[Text Word] OR diagnostic decision support[Text Word] OR personal health record decision support[Text Word] OR decision support systems[Text Word] OR electronic health records decision support[Text Word] OR electronic medical records decision support[Text Word] OR clinical decision rule[Text Word] OR infobutton[Text Word] OR reminder[Text Word] OR order set[Text Word] OR computerized physician order entry[Text Word] OR computerized provider order entry[Text Word] OR computerized provider order management[Text Word] OR Medical Order Entry System[Text Word] OR dashboard[Text Word] OR Decision Support Systems, Clinical[MeSH Major Topic] OR Expert Systems[MeSH Major Topic] OR Decision Support Techniques[MeSH Major Topic] OR Clinical Decision Rules[MeSH Major Topic] OR Medical Order Entry Systems[MeSH Major Topic]) AND (artificial intelligence[Text Word] OR machine learning[Text Word] OR predictive model[Text Word] OR neural network[Text Word] OR deep learning[Text Word] OR natural language processing[Text Word] OR NLP[Text Word] OR fuzzy logic[Text Word] OR ontology[Text Word] OR knowledge graph[Text Word] OR Computer Heuristics[MeSH Major Topic] OR Fuzzy Logic[MeSH Major Topic] OR Machine Learning[MeSH Major Topic] OR Natural Language Processing[MeSH Major Topic] OR Biological Ontologies[MeSH Major Topic] OR Neural Networks, Computer[MeSH Major Topic] OR Robotics[MeSH Major Topic] OR Deep Learning[MeSH Major Topic] OR Artifical Intelligence[MeSH Major Topic]) NOT (“dataset”[Publication Type] OR “review”[Publication Type] OR "systematic review"[Publication Type] OR "meta analysis"[Publication Type] OR "case reports"[Publication Type] OR "comment”[Publication Type] OR "editorial"[Publication Type] OR "news"[Publication Type] OR "English abstract"[Publication Type] OR "congress"[Publication Type] OR "overall"[Publication Type])*

Search fields: *“Text Word”, “MeSH Major Topic”*

Filters: Years including and before 2022.

**2. EMBASE**

Search string:

*(pregnancy OR Fetus OR Infant OR Newborn OR baby OR pregnant OR maternal OR preterm OR stillbirth OR miscarriage OR abortion OR fetal OR birth OR labor OR perinatal OR prenatal OR antenatal OR gestational OR gestation OR postpartum OR Obstetrics OR Gynecology OR genital OR reproductive OR Pregnancy OR Pregnancy Outcome OR Fetus OR Infant OR Infant, Newborn OR Neonatal Screening OR Infant Mortality OR Fetal Death OR Infant Death OR Perinatal Death OR Perinatal Mortality OR Abortion, Habitual OR Embryo Loss OR Fetal Viability OR Prenatal Diagnosis OR Postpartum Period OR Depression, Postpartum OR Prenatal Care OR Premature Birth OR Infant, Premature OR Obstetric Labor, Premature OR Infant, Extremely Premature OR Infant, Extremely Low Birth Weight OR Gynecology OR Obstetrics OR Fetal Monitoring OR Pregnancy Complications OR Maternal Mortality) AND (clinical decision support OR decision aid OR diagnostic decision support OR personal health record decision support OR decision support systems OR electronic health records decision support OR electronic medical records decision support OR clinical decision rule OR infobutton OR reminder OR order set OR computerized physician order entry OR computerized provider order entry OR computerized provider order management OR Medical Order Entry System OR dashboard OR Decision Support Systems, Clinical OR Expert Systems OR Decision Support Techniques OR Clinical Decision Rules OR Medical Order Entry Systems) AND (artificial intelligence OR machine learning OR predictive model OR neural network OR deep learning OR natural language processing OR NLP OR fuzzy logic OR ontology OR knowledge graph OR Computer Heuristics OR Fuzzy Logic OR Machine Learning OR Natural Language Processing OR Biological Ontologies OR Neural Networks, Computer OR Robotics OR Deep Learning OR Artifical Intelligence)*

Search fields: *“title, abstract, or author keywords”*

Filters: Years including and before 2022.

**3. ACM Digital Library**

Search string:

*(pregnancy OR Fetus OR Infant OR Newborn OR baby OR pregnant OR maternal OR preterm OR stillbirth OR miscarriage OR abortion OR fetal OR birth OR labor OR perinatal OR prenatal OR antenatal OR gestational OR gestation OR postpartum OR Obstetrics OR Gynecology OR genital OR reproductive OR Pregnancy OR Pregnancy Outcome OR Fetus OR Infant OR Infant, Newborn OR Neonatal Screening OR Infant Mortality OR Fetal Death OR Infant Death OR Perinatal Death OR Perinatal Mortality OR Abortion, Habitual OR Embryo Loss OR Fetal Viability OR Prenatal Diagnosis OR Postpartum Period OR Depression, Postpartum OR Prenatal Care OR Premature Birth OR Infant, Premature OR Obstetric Labor, Premature OR Infant, Extremely Premature OR Infant, Extremely Low Birth Weight OR Gynecology OR Obstetrics OR Fetal Monitoring OR Pregnancy Complications OR Maternal Mortality) AND (clinical decision support OR decision aid OR diagnostic decision support OR personal health record decision support OR decision support systems OR electronic health records decision support OR electronic medical records decision support OR clinical decision rule OR infobutton OR reminder OR order set OR computerized physician order entry OR computerized provider order entry OR computerized provider order management OR Medical Order Entry System OR dashboard OR Decision Support Systems, Clinical OR Expert Systems OR Decision Support Techniques OR Clinical Decision Rules OR Medical Order Entry Systems) AND (artificial intelligence OR machine learning OR predictive model OR neural network OR deep learning OR natural language processing OR NLP OR fuzzy logic OR ontology OR knowledge graph OR Computer Heuristics OR Fuzzy Logic OR Machine Learning OR Natural Language Processing OR Biological Ontologies OR Neural Networks, Computer OR Robotics OR Deep Learning OR Artifical Intelligence)*

Search fields: *“title”, “abstract”, and “author keyword”*

Filters: Years including and before 2022.
